# Clinicopathological and prognostic significance of LINC00673 in human malignancy: a review and meta-analysis

**DOI:** 10.1042/BSR20211175

**Published:** 2021-07-28

**Authors:** Yurong Zhu, Zhifa Zhang, Hui Peng, Weiping Li, Shaowei Hu, Min Zhao, Weifeng Lin

**Affiliations:** 1 Department of Pathology, Dongguan People’s Hospital (Affiliated Dongguan People’s Hospital, Southern Medical University), Dongguan, China; 2 Department of Pathology, Guangdong Provincial Hospital of Chinese Medicine, The Second Clinical Medical College, Guangzhou University of Chinese Medicine, Guangzhou, China; 3 Department of Pathology, Guangdong Provincial Hospital of Chinese Medicine-Zhuhai Hospital, Zhuhai, China; 4 Department of Neurology, The First Affiliated Hospital of Jinan University, Guangzhou, China; 5 Department of Neurology, Dongguan People’s Hospital (Affiliated Dongguan People’s Hospital, Southern Medical University), Dongguan, China

**Keywords:** Linc00673, malignant tumour, meta-analysis, prognosis

## Abstract

**Background:** We conducted this research to investigate the relationship between long intergenic non-protein coding RNA 673 (linc00673) expression and prognosis and clinicopathological parameters in human malignancies.

**Methods:** The PubMed, Embase, WOS, and CNKI databases were used to collect eligible research data before 4 January 2021. Meta-analysis was performed using Stata 12.0 software. Pooled odds ratios (ORs) or hazard ratios (HRs) and their 95% confidence interval (CIs) were calculated to evaluate the association of linc00673 expression with survival outcomes and clinical parameters.

**Results:** We finally included 17 articles and a total of 1539 cases for the meta-analysis. The results indicated that linc00673 was significantly correlated with T stage (*P*=0.006), tumor stage (*P*<0.001), lymph node metastasis (*P*<0.001), and distant metastasis (*P*<0.001). In addition, the results suggested that elevated linc00673 expression predicted a poor overall survival (OS) time (*P*=0.013) and acted as an independent prognostic factor (*P*<0.001) for OS in patients with malignancy. Although potential evidence of publication bias was found in the studies on OS in relation to tumor stage in the multivariate analysis, the trim-and-fill analysis confirmed that the results remained stable.

**Conclusions:** Overexpression of linc00673 was significantly correlated with shorter OS time in patients with malignant tumors. Moreover, the increased expression level of linc00673 was significantly correlated with T stage, tumor stage, lymph node metastasis, and distant metastasis. The results presented in this article revealed that linc00673 might be involved in the progression and invasion of malignancy and serve as a novel prognostic biomarker and potential therapeutic target for malignancy.

## Introduction

Malignant neoplasms are a complex, multifactorial and worldwide public health problem, with increasing incidence and mortality in this century [1]. Human cancers arise from the stepwise accumulation of genetic and epigenetic alterations [2]. In previous studies, intense efforts have been devoted to understanding proteins and their functions, and RNA is understood to be a mediator of the translation of the genetic code from DNA to protein. In recent years, with the rapid advance of high-throughput sequencing technologies, multiple studies have revealed that although over 75% of the human genome is transcribed [3], only approximately 2% of it encodes proteins [4]. Most RNA transcripts are not translated into proteins and are referred to as non-coding RNAs (ncRNAs).

Depending on the number of nucleotides, ncRNAs are classified as small ncRNAs (sncRNAs) and long ncRNAs (lncRNAs). LncRNAs are a large group of transcripts that are >200 nucleotides in length and are categorized as intronic lncRNAs, intergenic lncRNAs (lincRNAs), enhancer lncRNAs (elncRNAs), bidirectional lncRNAs, overlapping lncRNAs, and antisense lncRNAs on the basis of their genomic localization [5]. LincRNAs are likely the most important group of lncRNAs and originate from the region between two protein-coding genes and were previously regarded as ‘junk’ DNA [6]. Of note, however, increasing evidence suggests that lncRNAs may play important roles in facilitating and maintaining cancer origination and progression [5,7,8] by interacting with chromosomes, RNA, and proteins [9].

Among these tumor-related lincRNAs, a newly discovered lincRNA referred to as ‘long intergenic non-protein coding RNA 673’ (linc00673), also known as ‘SRA-like non-coding RNA’ (SLNCR) or ERRLR01, has drawn increasing attention. Linc00673 is located on chromosome 17 and has a total of five transcript variants (V1, V2, V3, V4, and V5), as shown in the NCBI database. Some studies have shown that linc00673 expression is down-regulated in pancreatic cancer, and as a suppressor, linc00673 overexpression may inhibit the proliferation and migration of cancer cells [10,11]. However, multiple studies have revealed that the expression of linc00673 is markedly elevated in other human cancer tissues and cells [12,13]. It has been described that linc00673 promotes tumor progression via multiple pathways, including anti-apoptosis, proliferation, metastasis and invasiveness pathways, and promotes cancer stem-like cell properties [14,15]. Linc00673 may indicate poor prognosis of malignancies [16]. In brief, there is a high level of discrepancy in the literature regarding the role of linc00673 expression. Therefore, a systematic review and quantitative meta-analysis that aimed to investigate the clinicopathological and prognostic value of linc00673 as a potential biomarker in human malignant tumors was conducted.

## Materials and methods

### Retrieval strategy and registration

**Figure 1:**
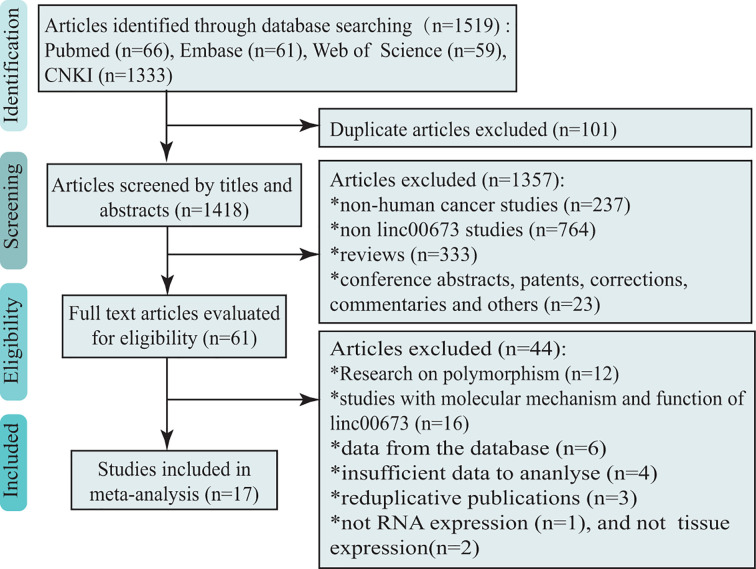
Flow chart of search strategy and study selection

An extensive electronic search of the literature was conducted through databases including PubMed, Web of Science, Embase, and CNKI for eligible studies published until 4 January 2021, with the following terms: ‘(neoplasms OR neoplasia OR carcinoma OR tumour OR cancer OR malignancy OR epithelioma OR sarcoma OR lymphoma OR leukaemia) and (LINC00673 OR long intergenic nonprotein coding RNA 673 OR LncRNA00673 OR SLNCR1 OR SLNCR OR ERRLR01)’. The search language, publication year, and race were not restricted. To find additional studies, we manually screened the reference lists of review articles. A flowchart of the study screening process is shown in Figure 1. This project was registered in PROSPERO (ID: CRD42020149189).

### Quality assessment

Methodological quality evaluation of the selected studies was performed independently by two investigators. Any discrepancies found in the included studies were resolved through discussion or consultation with a third investigator until a consensus was reached. The Newcastle–Ottawa Quality Assessment Scale (NOS) was used to assess the quality of each cohort study (http://www.ohri.ca/programs/clinical_epidemiology/oxford.asp), while the Agency for Healthcare Research and Quality (AHRQ) was used to assess the quality of cross-sectional observational studies [17] (Table 1). NOS scores were subdivided into those indicating high quality (≥6) and low quality (<6) [18], and AHRQ scores were subdivided into those indicating high quality (8–11), moderate quality (4–7), and low quality (0–3).

**Table 1:** Basic characteristics and quality assessment of the included studies

Study	Year	Tumor type	Detection method	Cut-off	Sample size	*n* (high/low)^1^	Expression in tumor (x ± SD/rate, *P*)^2^	AHRQ score	NOS score	HR statistic	Clinicopathological parameters included in the meta-analysis
Feng	2018	CRC	qRT-PCR	Fold change > 4.5	71	36/35	Up (71.8%, NA)	NA	8	Data in paper	Age, gender, tumor size, TS (TNM), LNM, DM, TD
Yang	2017	PDAC	qRT-PCR	Mean value	50	25/25	Down (NA, *P*=0.003)	NA	7	Survival curve	Age, gender, tumor size, T stage, LNM, TD
Tan	2017	NSCLC	qRT-PCR	Median level	76	38/38	Up (NA, *P*<0.01)	6	NA	NA	Age, gender, tumor size, T stage, LNM, TD
Shi	2020	CC	qRT-PCR	NA	63	31/32	Up (4.26 ± 0.75, *P*<0.001)	NA	9	Data in paper	Age, tumor size, TS (FIGO), LNM, TD
Qiao	2019	BC	qRT-PCR	NA	80	40/40	Up (NA, *P*<0.0001)	NA	9	Survival curve	Age, tumor size, TS (TNM), LNM
Zhou	2020	ESCC	RNA FISH	Score > 1	81	34/47	Up (NA, *P*<0.0001	NA	9	Survival curve	Age, gender, tumor size, T stage, TS (TNM), TD,
Gong	2020	PC	qRT-PCR	NA	57	28/29	Down (NA, *P*<0.001)	NA	7	Survival curve	NA
Xia	2018	TC	qRT-PCR	Median value	60	30/30	Up (NA, *P*<0.05)	5	NA	NA	Age, gender, tumor size, TS (AJCC), LNM
Ba	2017	GC	qRT-PCR	Median	79	46/33	Up (NA, *P*<0.05)	NA	8	Data in paper	Age, gender, tumor size, T stage, TS (TNM), LNM, DM, TD
Huang	2017	GC	qRT-PCR	Fold-change ≥ 2	73	30/43	Up (NA, *P*<0.01)	NA	8	Data in paper	Age, gender, tumor size, TS (TNM), LNM, TD
Zheng	2019	EOC	qRT-PCR	Median value	131	66/65	Up (NA, *P*<0.0001)	NA	8	Survival curve and Data in paper	Age, tumor size, TS (FIGO), LNM, TD
Guan	2019	LAD	qRT-PCR	ROC curve analysis	119	76/43	Up (NA, *P*<0.001)	NA	9	Survival curve	Age, gender, T stage, TS, LNM, DM
Zhang	2018	PC	qRT-PCR	NA	229	NA	Up (NA, *P*<0.05)	NA	8	Lack of data	Age, TS, LNM, DM, TD
Xia	2018	BC	qRT-PCR	median value	35	22/13	Up (NA, *P*<0.05)	4		NA	Age, tumor size, TS, LNM
Zhang	2017	HCC	qRT-PCR	median	53	37/16	Up (NA, *P*<0.01)	NA	8	Survival curve	NA
Yu	2017	TSCC	ISH	SQSC	202	110/92	Up (NA, *P*=0.020)^3^	NA	8	Data in paper	Age, gender, T stage, TS (TNM), LNM, DM
Shi	2016	NSCLC	qRT-PCR	ROC curve analysis	80	41/39	Up (NA, *P*<0.05)	5		NA	Age, gender, tumor size, TS (TNM), LNM, TD

Abbreviations: BC, breast cancer; CC, cervical cancer; CRC, colorectal cancer; DM, distant metastasis; EOC, epithelial ovarian cancer; ESCC, oesophageal squamous cell carcinoma; GC, gastric cancer; HCC, hepatocellular carcinoma; LAD, lung adenocarcinoma; LNM, lymph node metastasis; NSCLC, non-small cell lung cancer; PC, pancreatic cancer; PDAC, pancreatic ductal adenocarcinoma; ROC, receiver operating characteristic curve; SQSC, semi-quantitative scoring criterion; TC, thyroid carcinoma; TD, tumor differentiation; TS, tumor stage; TSCC, tongue squamous cell carcinoma.

^1^The number of high- and low-expression of LINC00673. ^2^LINC00673 expression in tumors compared with control group. ^3^The sample size is 15.

### Inclusion and exclusion criteria

The inclusion criteria were as follows: (i) only patients with malignant tumors were included in the present study; (ii) linc00673 RNA expression was assessed in tissues, not plasma or other fluids, by valid techniques; (iii) clinicopathologic parameters such as lymph node metastasis, tumor differentiation, tumor stage and distant metastasis were described, and (iv) the association between linc00673 and the overall survival (OS) time was evaluated, for which the hazard ratios (HRs) and 95% confidence interval (CIs) or Kaplan–Meier curves were calculated.

The exclusion criteria were as follows: (i) reviews, case reports, conference abstracts, patents, corrections, and commentaries; (ii) reduplicative publications; (iii) studies focused on other lncRNAs or linc00673 polymorphisms; (iv) studies that only concentrated on the function of linc00673 and possible molecular mechanisms, such as experiments on cells and animals, and (v) studies for which data were unavailable.

Data were extracted independently by two researchers and were thoroughly reviewed and discussed. Specifically, the following information was extracted from each study: the surname or full name of first author, the year of publication, the name of the journal or other publication, the language of the publication, the country the publication originated from, the patient tumor type evaluated, the detection method used, the sample size, the cut-off value of linc00673 expression, linc00673 expression in patients with malignant tumor, patients’ age and sex, tumor size, the T stage, tumor stage, lymph node metastasis and distant metastasis status, the tumor differentiation status and the OS rate. If we could not extract HRs and 95% CIs of the OS rate directly from the articles, Kaplan–Meier survival curves were collected for further computation using Engauge Digitizer software per the protocol described by Bennounna et al. and Tierney et al. [19,20].

### Statistical analyses

All statistical analyses were performed with Stata 12 software (Stata Corp, College Station, Texas, U.S.A.) by the investigator. Pooled HRs and ORs were used for the evaluation of OS rates and clinicopathologic parameters, respectively. Interstudy heterogeneity was investigated by the chi-square Q-test and *I^2^* statistic. *P*-values of the Q test (*P*_Q_) <0.05 and *I^2^* > 50% were considered to indicate significant heterogeneity. When there was obvious heterogeneity, a random-effects model was applied for statistical analyses, otherwise, a fixed-effects model was implemented. In addition, publication bias was examined after using Begg’s funnel plots and Egger’s linear regression tests, and trim-and-fill analysis was used to assess the impact of potential publication bias [21]. Sensitivity analysis was conducted to determine whether any single study could influence the overall result. *P*<0.05 was considered statistically significant.

## Results

### Study selection

After a preliminary online search, 1519 studies in total were originally retrieved from the electronic databases; 101 articles were repeats, and 1357 articles were excluded after reading the titles and abstracts. A total of 61 articles were identified for full-text screening, of which 17 were included for data extraction. The literature screening process is shown in Figure 1.

### General characteristics of the study populations

Finally, 17 articles [10–14,16,22–32] comprising 1539 patients were selected for the meta-analysis. All of the included studies were performed in China and were published in English from 2016 to 2020. The median sample size was 76, with a wide range from 35 to 229. Tumor types included lung cancer (three articles) [16,23,32], pancreatic cancer (three articles) [10,11,30], gastric cancer (two articles) [27,31], breast cancer (two articles) [12,14], colorectal cancer [22], cervical cancer [24], oesophageal squamous cell carcinoma [25], thyroid carcinoma [26], epithelial ovarian cancer [29], hepatocellular carcinoma [13], and tongue squamous cell carcinoma [31]. The basic characteristics of the included studies are presented in Table 1.

Data were extracted from 17 studies, of which 15 [10,12–14,16,22–30,32] contained information about the association between linc00673 and clinicopathologic parameters in a total of 1429 cancer patients. The clinicopathological parameters included age, sex, tumor size, T stage, tumor stage, lymph node metastasis, distant metastasis, and tumor differentiation (data shown in Supplementary Table S1). Meanwhile, 13 articles [10,11,13,14,16,22,24,25,27–31] contained information about the association between linc00673 expression and the OS rate, of which one study [30] lacked sufficient information to calculate the HR and 95% CI (data shown in Supplementary Table S2).

### Quality assessment

We utilized the NOS for cohort studies to assess the quality of the results presented in the articles. According to the criteria, all included studies were high-quality studies. Four studies involved only a cross-sectional measurement and were assessed using the AHRQ form. All of these studies were moderate-quality studies. The quality scores for the included studies are shown in Table 1.

### Meta-analyses of OS

**Figure 2:**
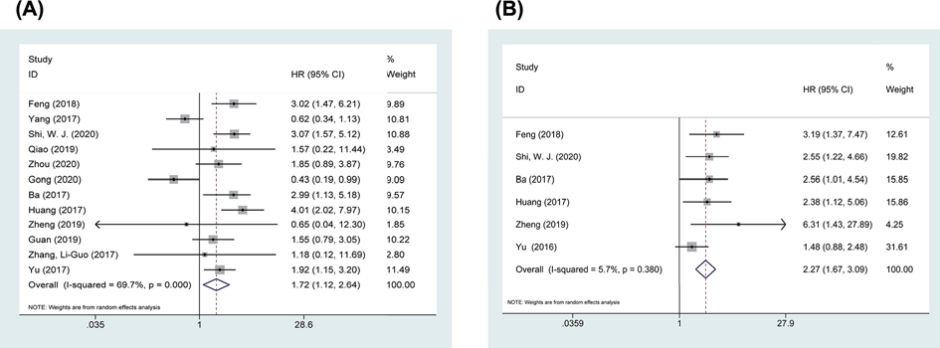
Forest plots of OS (**A**) Univariate OS; (**B**) multivariate OS.

The OS outcomes are shown in Table 2 and Figure 2. In the pooled analysis of 12 studies including 1059 patients, using a random-effects model because of data heterogeneity (*I^2^* = 69.7%, P_Q_ < 0.001) among the studies, we were surprised to find that linc00673 overexpression was obviously correlated with poor OS rates in patients with malignant neoplasms with a combined HR of 1.720 (95% CI: 1.120, 2.643; *P*=0.013) (Figure 2A). Furthermore, a multivariate pooled analysis of six studies with 619 patients was conducted to explore the independent prognostic value of linc00673 expression in different malignancies. The pooled HRs revealed that the expression of linc00673 was an independent prognostic risk factor for OS (HR = 2.271, 95% CI: 1.668, 3.093, *P*<0.001) with no heterogeneity (*I^2^* = 5.7%, P_Q_ = 0.380) (Figure 2B).

**Table 2:** Association between linc00673 expression and the OS time

Survival analysis	N	Patients (*n*)	HR	95% CI	*P*	Heterogeneity (*I^2^*, P_Q_)	Model
Univariate K-M	12	1059	1.720	1.12, 2.643	0.013	69.7%, <0.001	Random
Multivariate K-M	6	619	2.271	1.668, 3.093	<0.001	5.7%, 0.380	Fixed

If *I^2^* > 50%, the results were calculated by random model.

N, number of included studies.

### Meta-analyses of clinicopathological parameters

**Figure 3:**
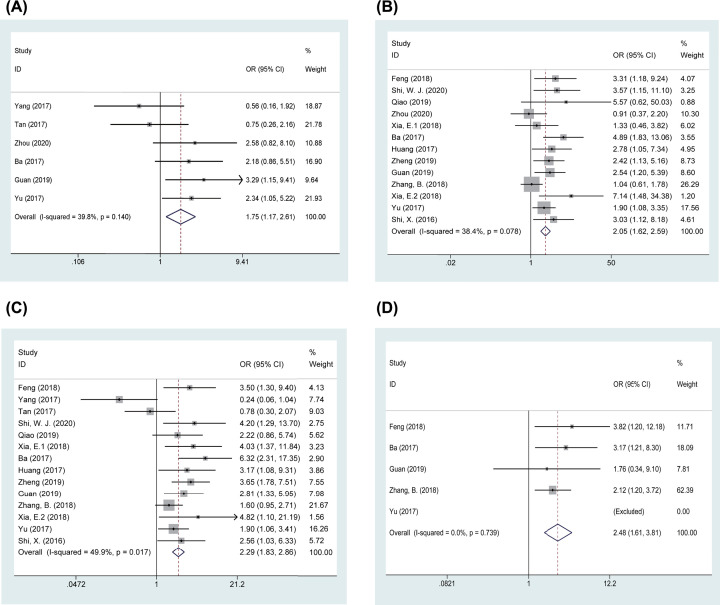
Forest plots of clinicopathological parameters (**A**) T stage; (**B**) tumor stage; (**C**) lymph node metastasis; (**D**) distant metastasis.

The clinicopathological parameter outcomes are shown in Table 3 and Figure 3. Using the chi-square Q test and *I^2^* test, heterogeneity was observed between linc00673 overexpression and sex (*I^2^* = 51.3%, P_Q_ = 0.030) and tumor size (*I^2^* = 73.4%, P_Q_ < 0.001); thus, a random-effects model was applied. The increased expression level of linc00673 was significantly associated with T stage (pooled odds ratio (OR) = 1.749, 95% CI: 1.171, 2.612, *P*=0.006) (Figure 3A), tumor stage (pooled OR = 2.047, 95% CI: 1.621, 2.585, *P*<0.001) (Figure 3B), lymph node metastasis (pooled OR = 2.288, 95% CI: 1.830, 2.862, *P*<0.001) (Figure 3C), and distant metastasis (pooled OR = 2.479, 95% CI: 1.611, 3.813, *P*<0.001) (Figure 3D). There was no significant association between linc00673 overexpression and patient age (*P*=0.800) (Supplementary Figure S1A), sex (*P*=0.861) (Supplementary Figure S1B), tumor size (*P*=0.196) (Supplementary Figure S1C), or tumor differentiation (*P*=0.112) (Supplementary Figure S1D).

**Table 3:** Association between linc00673 and clinicopathological parameters

Clinicopathological parameters	N	Patients (*n*)	OR	95% CI	*P*	Heterogeneity (*I^2^*, P_Q_)	Model
Age (elderly vs. nonelderly)	15	1429	0.973	0.787, 1.203	0.800	0.0%, 0.725	Fixed
Gender (male vs. female)	10	891	1.041	0.667, 1.625	0.861	51.3%, 0.030	Random
Tumor size (larger vs. smaller)	12	879	1.493	0.813, 2.741	0.196	73.4%, <0.001	Random
T stage (later vs. earlier)	6	607	1.749	1.171, 2.612	0.006	39.8%, 0.140	Fixed
Tumor stage (later vs. earlier)	13	1303	2.047	1.621, 2.585	<0.001	38.4%, 0.078	Fixed
Lymph node metastasis (positive vs. negative)	14	1348	2.288	1.830, 2.862	<0.001	49.9%, 0.017	Fixed
Distant metastasis (presence vs. absence)	5	700	2.479	1.611, 3.813	<0.001	0.0%, 0.739	Fixed
Tumor differentiation (poor vs. well, moderate)	10	933	1.268	0.946, 1.699	0.112	47.4%, 0.047	Fixed

If *I^2^* > 50%, the results were calculated by a random model.

N, number of included studies.

### Publication bias and sensitivity analysis

**Figure 4:**
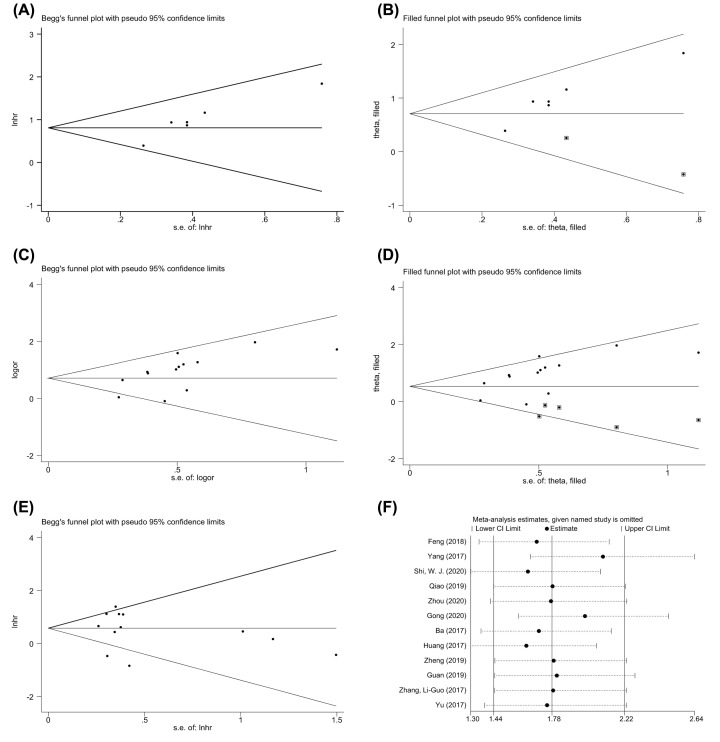
Publication bias and sensitivity analysis in this meta-analysis (**A,B**) Publication bias for multivariate OS. (**C,D**) Publication bias for tumor stage. (**E**) Publication bias for univariate OS. (**F**) Sensitivity analysis for univariate OS.

Begg’s funnel plot and Egger’s regression test were used to determine publication bias. Begg’s test (*P*=0.244) and Egger’s test (*P*=0.667) illustrated the absence of publication bias for OS in the univariate analysis (Supplementary Table S3, Figure 4E). For OS in the multivariate analysis, the visual measurement of the funnel plot exhibited slight asymmetry (Figure 4A), and Egger’s test (*P*=0.006) revealed probable evidence of publication bias (Supplementary Table S3). Furthermore, to explore the impact of potential publication bias, we performed a trim-and-fill analysis with a fixed-effects model. The study was filled with two other studies by three iterations (Figure 4B). Likewise, linc00673 expression was an independent prognostic factor for worse OS rates in patients with malignancy (HR (filled) = 2.033, 95% CI: 1.543, 2.678, *P*<0.001). These results suggested that the effect of publication bias was not significant, and that the conclusion was relatively stable.

No significant publication bias was observed in the clinicopathological parameters (Supplementary Table S3 and Figure S2A–G), except for tumor stage (*P*=0.019) (Supplementary Table S3, Figure 4C). To resolve this issue, the same trim-and-fill method was used. After analysis, the study was filled with five other studies by five iterations (Figure 4D). The pooled analysis included hypothetical studies and continued to indicate a statistically significant association between tumor stage and the expression level of linc00673 (filled OR = 1.754, 95% CI 1.266, 2.431, *P*=0.001). The result was the same as previously obtained, which indicated that the effect of publication bias was not significant and that the conclusion was stable.

A sensitivity analysis was performed in which one study was omitted at a time to explore the effect of the excluded dataset on each clinicopathological parameter and the OS rate. The relationship continued to be significant when any single study was removed, verifying the stability of the conclusion (Figure 4F, Supplementary Figure S3A–I).

## Discussion

In pancreatic cancer, the earliest report on linc00673, newly discovered gene locus, revealed that this lincRNA is significantly associated with the risk for cancer as determined by a genome-wide association study (GWAS) [33]. On one hand, it was demonstrated that linc00673 expression was significantly down-regulated in pancreatic cancer tissues and cell lines and that it acts as a novel tumor suppressor that can inhibit cancer cell cycle progression, proliferation and tumorigenesis; silencing linc00673 can promote cell proliferation [10,11,34]. On the other hand, as more in-depth research has been conducted, it has been found that linc00673 is enriched in a wide variety of tumors and plays an important role in tumorigenesis and tumor biological behavior, including cell proliferation, migration, invasion, and tumor metastasis [35]. First, linc00673 regulates the invasion and metastasis capabilities of breast cancer cells by regulating B7-H6 expression [12]. Second, it can also act as a scaffold for Wnt/β-catenin signalling which, when overactivated, promotes lung adenocarcinoma cell migration, invasion, and metastasis [16]. Third, linc00673 is known to form a complex with androgen receptor (AR), and Brn3a activates matrix metalloproteinase 9 (MMP9), which has been shown to increase melanoma invasiveness [36]. Moreover, linc00673 and AR might act together to regulate p21 (a primary inhibitor of cyclin-dependent kinases) and subsequently enhance melanoma cell proliferation and tumor growth [37]. Furthermore, linc00673 plays a competing endogenous RNAs (ceRNA) role in regulating gene expression by competitively binding to miRNAs, such as miR-150-5p [35] and miR-515-5p [14], eventually leading to proliferation, migration, invasion, and Epithelial-Mesenchymal Transition (EMT).

In clinical tests, a growing body of data has revealed the relationship between linc00673, clinicopathological parameters and the prognosis of various malignancies. It has been observed that linc00673 overexpression is correlated with poor prognosis of patients with several types of tumor in many studies [16,25,28]. However, the results of other studies have indicated that higher linc00673 expression may indicate a better prognosis [10,11]. Some articles have shown that the levels of linc00673 do not correlate with any of the clinicopathological parameters [23]. Yang et al. investigated linc00673 expression down-regulation in pancreatic ductal adenocarcinoma (PDAC), and the results showed that high linc00673 expression was associated with smaller tumor size, a lower frequency of lymph node involvement, and good tumor differentiation [10]. However, many other studies have revealed that linc00673 overexpression correlates positively with the aggressive clinicopathological features of malignant tumors [14,28,31,32]. Overall, the relationship between linc00673 expression and clinicopathological features, as well as its influence on prognostic outcome, remain under dispute. To provide clarity, we are the first to combine all published studies in a meta-analysis to evaluate the clinical and prognostic values of linc00673 among different types of malignancies. Above all, the results of the present study showed that patients with high linc00673 expression had a worse OS rates in both the univariate and multivariate survival analyses, suggesting that linc00673 was an independent prognostic factor in patients with malignancy. These results were similar to the previous meta-analysis by Gao et al [38]. They also reported that linc00673 demonstrated significantly prognostic value. Similarly, Zhu et al. in his literature review article explored that the high expression of linc00673, which acts as a pro-oncogene, is related to adverse outcomes for patients [39]. Moreover, our results showed that in patients with malignancy, the increased expression of linc00673 was associated with later T stage and tumor stage and an increased likelihood of lymph node metastasis and distant metastasis. These results suggest a role for linc00673 in the invasiveness and metastasis of malignancies.

This meta‐analysis had several limitations. First, the number of samples examined in the present study was relatively small, which might account for the heterogeneity across studies and publication bias related to tumor stage. Second, different detection methods of linc00673 were used in the included studies, and even when the same qRT-PCR assay methods were used, the heterogeneity among studies could be attributable to the use of different PCR primer sets. Third, no consensus on cut‐off values for high and low linc00673 expression among the studies was reached, and not all articles reported these data. Fourth, several original studies did not provide complete data to allow for inclusion in the analysis. Finally, potential heterogeneity may also be caused by other confounding factors, such as different tumor types and different study designs.

## Conclusion

In summary, this meta‐analysis revealed that overexpression of linc00673 was significantly correlated with a shorter OS time in patients with malignant tumors and might serve as a novel effective prognostic biomarker and potential therapeutic target for malignancy. Moreover, the increased expression level of linc00673 was significantly correlated with T stage, tumor stage, lymph node metastasis, and distant metastasis, which indicated that linc00673 is involved in the progression and invasion capabilities of malignancies. However, larger scale and deeper investigations and higher quality clinical studies across ethnicities are necessary to explore the prognostic value and tumorigenic role of linc00673 before its expression level can be used clinically.

## Supplementary Material

Supplementary Figures S1-S3 and Tables S1-S3

## Data Availability

All data generated or analyzed during the present study are included in this published article and its supplementary information files.

## Abbreviations

AHRQ: Agency for Healthcare Research and Quality
AR: androgen receptor
ceRNA: competing endogenous RNAs
CI: confidence interval
EMT: Epithelial-Mesenchymal Transition
HR: hazard ratio
lincRNA: long intergenic non-coding RNA
linc00673: long intergenic non-protein coding RNA 673
lncRNA: long non-coding RNA
ncRNA: non-coding RNA
NOS: Newcastle–Ottawa Quality Assessment Scale
OR: odds ratio
OS: overall survival
SLNCR: SRA-like non-coding RNA
