# Supplementary material for: Clinicopathological and prognostic significance of LINC00673 in human malignancy: a review and meta-analysis
Source: Biosci Rep. 2021 Jul 28;41(7):BSR20211175. doi: 10.1042/BSR20211175 (PMC8319490; doi:10.1042/BSR20211175)
Supplement: Supplementary Figures S1-S3 and Tables S1-S3 [file BSR-2021-1175_supp.pdf]

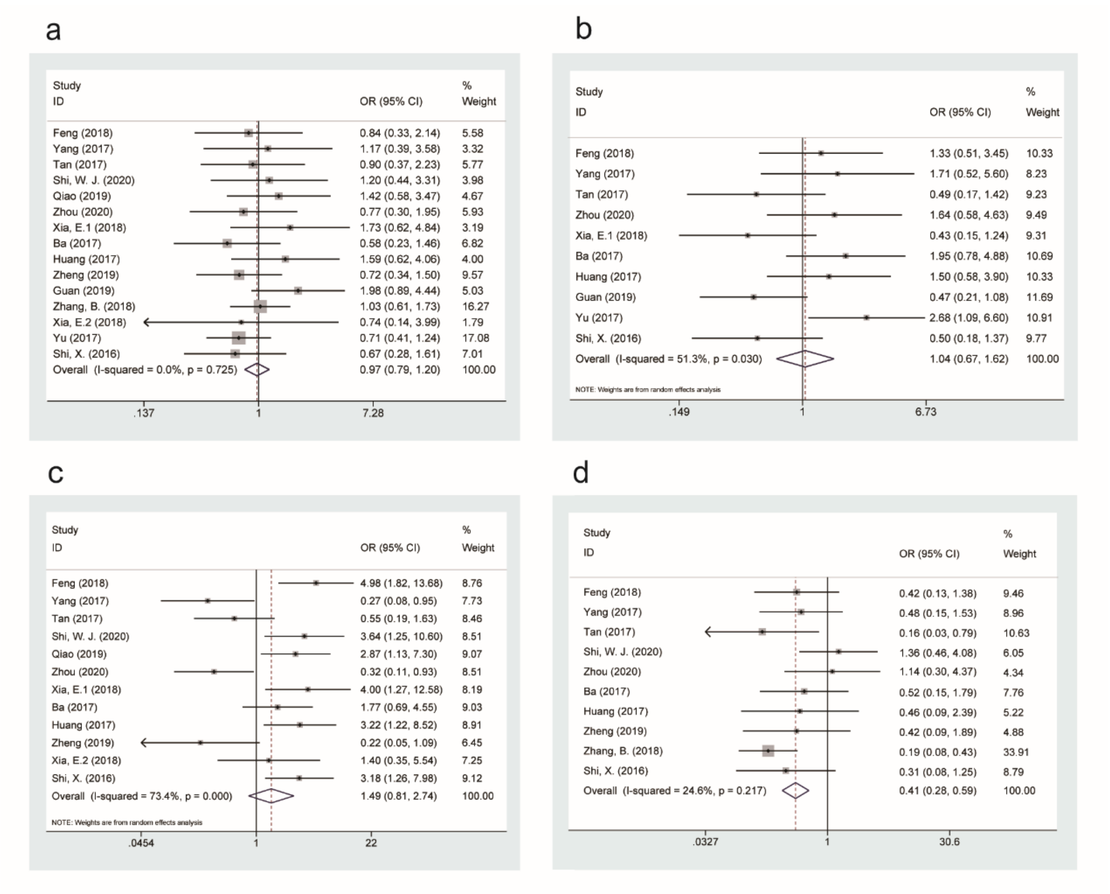

Figure S1. Forest plots of clinicopathological parameters. (a) Age; (b) Gender; (c) Tumor size; (d) Tumor differentiation.

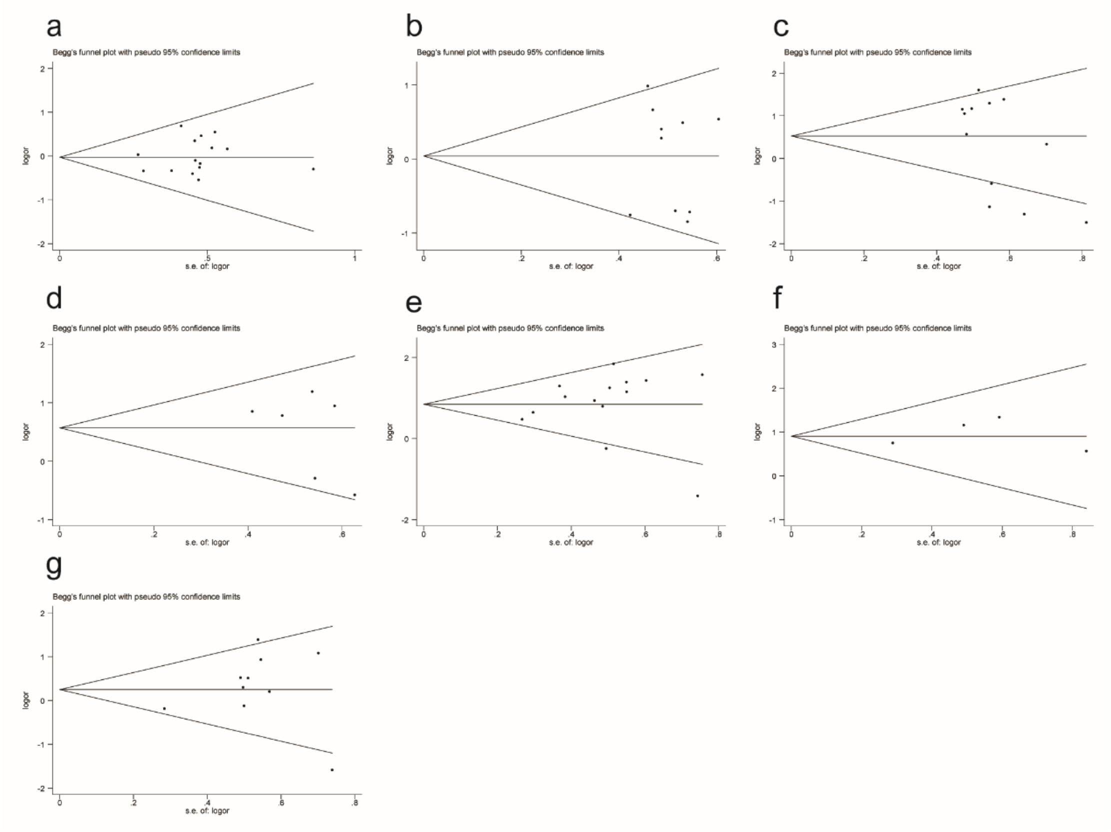

Figure S2. Publication bias in this meta-analysis. (a) Age; (b) Gender; (c) Tumor size; (d) T stage; (e) Lymph node metastasis; (f) Distant metastasis; (g) Tumor differentiation.

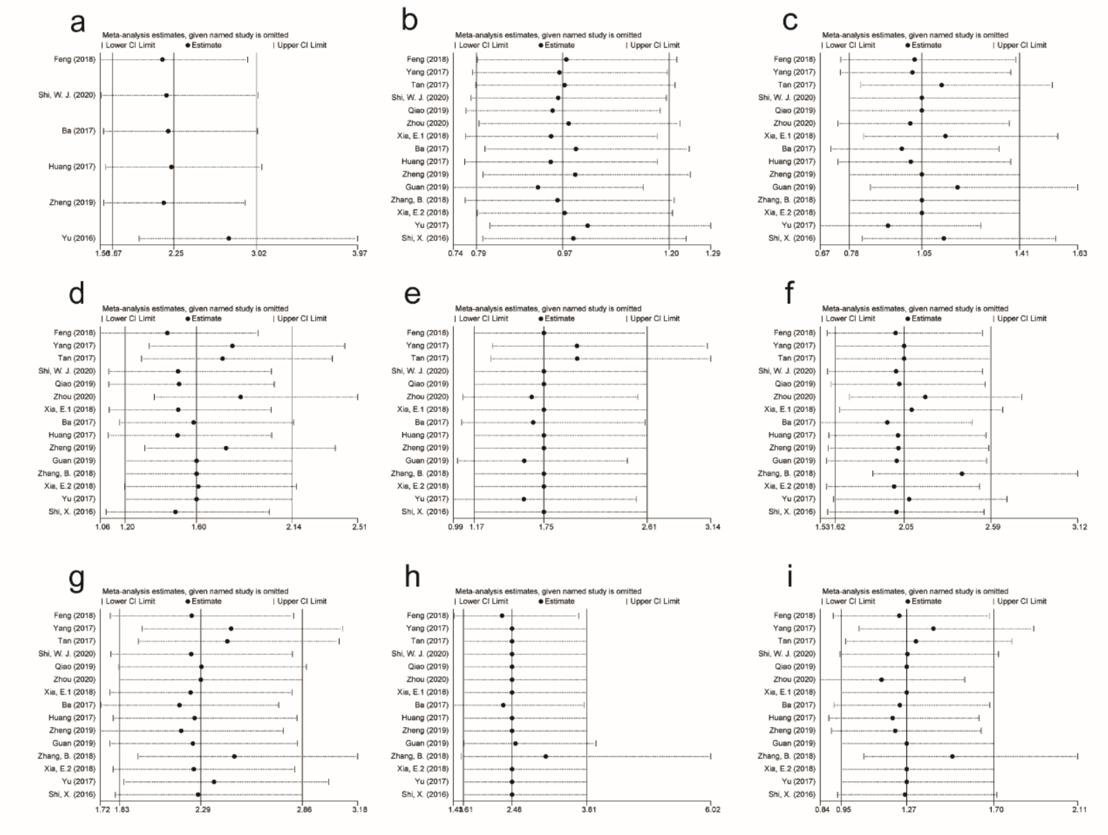

Figure S3. Sensitivity analysis in this meta-analysis. (a) Multivariate OS; (b) Age; (c) Gender; (d) Tumor size; (e) T stage; (f) Tumor stage; (g) Lymph node metastasis; (h) Distant metastasis; (i) Tumor differentiation.

Table SI Data extraction for the association between linc00673 expression and clinic-pathological features of malignancy

Study（year）	high_elderly	high_nonelderly	low_elderly	low_nonelderly	high_male	high_female	low_male	low_female	high_larger	high_smaller	low_larger	low_smaller	high_laterT	high_earlierT	low_laterT	low_earlierT	high_laterTS	high_earlierTS	low_laterTS	low_earlierTS	high_M1	high_M0	low_M1	low_M0	high_N1	high_N0	low_N1	low_N0	high_poor	high_midwell	low_poor	low_midwell
Feng (2018)	18	18	19	16	23	13	20	15	26	10	12	23					28	8	18	17	14	22	5	30	21	15	10	25	24	12	19	16
Yang (2017)	12	13	11	14	18	7	15	10	13	12	20	5	16	9	19	6									3	22	9	16	3	22	10	15
Tan (2017)	19	18	21	18	26	12	31	7	27	11	31	7	8	30	10	28									11	27	13	25	11	27	12	26
Shi, W. J. (2020)	13	18	12	20					17	14	8	24					14	17	6	26					14	18	5	27	9	22	8	24
Qiao (2019)	19	14	23	24					30	11	19	20					5	35	1	39					30	10	23	17				
Zhou (2020)	11	23	18	29	27	7	33	14	22	12	40	7	19	5	28	19	18	16	26	21									14	20	7	40
Xia, E.1 (2018)	19	11	15	15	9	21	15	15	24	6	15	15					12	18	10	20					21	9	11	19				
Ba (2017)	15	31	15	18	31	15	17	16	20	26	10	23	24	22	11	22	36	10	14	19	25	21	9	24	37	9	13	20	16	30	8	25
Huang (2017)	16	14	18	25	19	11	23	20	19	11	15	28					20	10	18	25					24	6	24	19	11	19	8	35
Zheng (2019)	43	23	47	18					2	64	8	57					51	15	38	27					44	22	23	42	63	3	57	8
Guan (2019)	32	43	12	32	19	15	62	23					9	8	26	76	35	30	17	37	3	41	3	72	35	18	27	39				
Zhang, B. (2018)	57	63	51	58													54	79	38	58	43	63	30	93	59	54	47	69	35	82	38	74
Xia, E.2 (2018)	4	18	3	10					12	10	6	7					15	7	3	10					15	7	4	9				
Yu (2017)	48	62	48	44	102	8	76	16					99	11	73	19	58	52	34	58	0	110	0	92	50	60	28	64				
Shi, X. (2016)	19	22	22	17	27	14	31	8	24	17	12	27					18	23	8	31					23	18	13	26	13	28	10	29

high (ref): linc00673 overexpression; low (ref): low expression of linc00673; M1 (ref): distant metastasis; M0 (ref): no distant metastasis; N1: lymph node metastasis; N0: no lymph node metastasis.

Table S2 Data extraction for the association between linc00673 expression and overall survival of malignancy

Study (year)	Univariate K-M				Multivariate K-M		
	HR	Lower 95% CI	Upper 95% CI		HR	Lower 95% CI	Upper 95% CI
Feng (2018)	3.023	1.471	6.213		3.194	1.365	7.471
Yang (2017)	0.622	0.341	1.135				
Shi, W. J. (2020)	3.066	1.566	5.118		2.552	1.224	4.662
Qiao (2019)	1.575	0.217	11.439				
Zhou (2020)	1.853	0.886	3.875				
Gong (2020)	0.433	0.190	0.991				
Ba (2017)	2.989	1.126	5.178		2.556	1.007	4.543
Huang (2017)	4.010	2.017	7.969		2.381	1.121	5.056
Zheng (2019)	0.653	0.035	12.303		6.309	1.427	27.888
Guan (2019)	1.549	0.787	3.050				
Zhang, Li-Guo (2017)	1.185	0.120	11.686				
Yu (2017)	1.924	1.155	3.205		1.478	0.881	2.479

K-M: Kaplan-Meier.

Table S3 The result of evaluated publication bias (*Begg* and *Egger* tests)

Parameter	N	Patients(n)	P		95%CI
			Begg`s	Egger`s	
Univariate OS	12	1059	0.244	0.667	-3.850, 2.573
Multivariate OS	6	619	0.060	0.006	1.518, 4.914
Age (elderly vs. nonelderly)	15	1429	0.322	0.523	-1.298, 2.430
Gender (male vs. female)	10	891	0.592	0.913	-12.045, 10.923
Tumor size (larger size vs. smaller size)	12	879	0.304	0.054	-14.426, 0.145
T stage (later vs. earlier)	6	607	0.260	0.318	-13.939, 5.828
Tumor stage (later vs. earlier)	13	1303	0.017	0.019	0.480, 4.328
Lymph node metastasis (positive vs. negative)	14	1348	0.443	0.631	-2.209, 3.501
Distant metastasis (presence vs. absence)	5	700	0.734	0.609	-3.607, 4.778
Tumor differentiation (poor vs. well, moderate)	10	933	0.858	0.464	-2.505-5.008

N: number of included studies.
